# Performances and Reliability of Bruker Microflex LT and VITEK MS MALDI-TOF Mass Spectrometry Systems for the Identification of Clinical Microorganisms

**DOI:** 10.1155/2015/516410

**Published:** 2015-12-17

**Authors:** Kivanc Bilecen, Gorkem Yaman, Ugur Ciftci, Yahya Rauf Laleli

**Affiliations:** ^1^Department of Research & Development, Duzen Laboratories Group, 06680 Ankara, Turkey; ^2^Department of Clinical Microbiology, Duzen Laboratories Group, 34387 Istanbul, Turkey; ^3^Department of Clinical Microbiology, Duzen Laboratories Group, 06680 Ankara, Turkey

## Abstract

In clinical microbiology laboratories, routine microbial identification is mostly performed using culture based methodologies requiring 24 to 72 hours from culturing to identification. Matrix assisted laser desorption ionization-time of flight mass spectrometry (MALDI-TOF MS) technology has been established as a cost effective, reliable, and faster alternative identification platform. In this study, we evaluated the reliability of the two available MALDI-TOF MS systems for their routine clinical level identification accuracy and efficiency in a clinical microbiology laboratory setting. A total of 1,341 routine phenotypically identified clinical bacterial and fungal isolates were selected and simultaneously analyzed using VITEK MS (bioMérieux, France) and Microflex LT (Bruker Diagnostics, Germany) MALDI-TOF MS systems. For any isolate that could not be identified with either of the systems and for any discordant result, 16S rDNA gene or ITS1/ITS2 sequencing was used. VITEK MS and Microflex LT correctly identified 1,303 (97.17%) and 1,298 (96.79%) isolates to the species level, respectively. In 114 (8.50%) isolates initial phenotypic identification was inaccurate. Both systems showed a similar identification efficiency and workflow robustness, and they were twice as more accurate compared to routine phenotypic identification in our sample pool. MALDITOF systems with their accuracy and robustness offer a good identification platform for routine clinical microbiology laboratories.

## 1. Introduction

Rapid and accurate identification of bacteria and yeasts from clinical specimens is crucial for the effective management of infections. In the clinical microbiology laboratories, microbial identification is conventionally done by phenotypic and biochemical analyses mostly using automated systems. These analyses require time ranging from a few hours to several days depending on microbial species in question. Workload and cost requirements for molecular methods, although they provide faster and accurate results, limit their routine use in clinical microbiology laboratories.

MALDI-TOF MS technology makes generation of unique mass spectral fingerprints of microorganisms possible, which are mostly a snapshot of ribosomal proteins ideal for an accurate microbial identification at the species level [1]. MALDI-TOF MS can rapidly and accurately identify a wide range of microorganisms at a reasonable cost using only a portion or the entire colony and a drop of matrix solution. While the MALDI-TOF MS analysis for the identification of intact bacterial cells was demonstrated 17 years ago [2, 3] and was extended to eukaryotic fungal cells 13 years ago [4, 5], not until recently has its potential for routine use been assessed for identification of a wide spectrum of bacteria, yeasts, molds, and mycobacteria that can be isolated in the clinical laboratories [6–13]. The ability of MALDI-TOF MS to directly identify bacteria in positive blood cultures is also important for the effective management of bloodstream infections [14, 15].

By this mean, two different CE marked in vitro diagnostic (IVD) MALDI TOF-MS systems were implemented to our laboratories in Ankara and Istanbul in March, 2012, and since then they have been used as the main identification tool for routine clinical bacterial and yeast isolates. In this study we evaluated performances of Bruker Microflex LT (Bruker Daltonics, Germany) and VITEK MS (bioMérieux, France) MALDI TOF-MS systems for identification of routine clinical microbiology isolates.

## 2. Materials and Methods

### 2.1. Clinical Isolates and Collection Strains

A total of 1,341 routine clinical isolates including 1,181 bacterial and 160 fungal specimens were analyzed using VITEK MS and Bruker Microflex LT. The isolates were obtained from clinical specimens including urine, blood, tissue, wounds, bronchoalveolar lavage, tracheal aspirate, sputum and wounds, which had been sent to our laboratory from various intensive care units, health facilities, and laboratories between April 2012 and December 2013. No selection criteria was applied for the isolates in order to obtain a comprehensive diversity.

Both MALDI-TOF MS systems were verified using 18 certified reference microorganisms obtained from reference culture collections.

### 2.2. Culture Conditions and Identification of Microorganisms

The specimens were routinely inoculated to appropriate media like, Columbia agar with 5% sheep blood, eosin methylene blue (EMB) agar, chromogenic urinary tract infection (CUTI) medium, chocolate agar, oxacillin resistant screening agar (ORSA),* Salmonella*-shigella agar, Thayer-Martin agar,* Candida* chromogenic agar, and blood culture bottles at 37°C depending on specimen type.

All of the specimens were incubated overnight at 37°C. The isolates were then Gram stained and identified using the reciprocate cards of VITEK II (bioMérieux, France) automated microbial identification system. The cultures and phenotypic identification results were transported to Ankara Duzen Laboratory Microbiology Department for VITEK MS analyses.

The strains were prepared and identified with VITEK MS system in Ankara and the results were documented. Following these initial identifications, culture plates were transported to our Clinical Laboratory Department located in İstanbul under appropriate conditions for the next day analysis with Bruker Microflex LT system.

### 2.3. VITEK MS MALDI-TOF System

From the overnight appropriate agar plates a portion or the whole colony was smeared onto the spots of VITEK MS-DS disposable target slides and then the spot was covered with 1 *μ*L of *α*-cyano-4-hidroxycinnamic acid (CHCA) matrix solution. Spots were completely air dried, and, then, the slides were placed on an adapter and inserted to VITEK MS instrument. Spectra were generated using the MYLA software (bioMérieux, France) and the identification was automatically done with the VITEK MS IVD version 2.4.0-5 software. This software version contains >25,000 spectra, covering 586 species consisting of 508 bacteria and 78 fungi in its database. The software compares the spectrum obtained to the expected spectrum of each organism or organism group. Then the percent probability, a quantitative value, is calculated for each sample. The range of percent probabilities for a correct identification is from 60 to 99 with values closer to 99.9 indicating a closer match. When the obtained percent probability is under 60, then it was considered as no-identification. Confidence level is determined with percent probability and number of choices. During our analyses, although there was a unique identification pattern within the good confidence level, a list of possible organisms was given for certain samples. In such cases if both organisms belonged to the same genus then this result was accepted as a reliable identification to the genus level. In some other cases, when the strain belonged to a complex (e.g.,* Enterobacter cloacae* complex) these results were documented as a correct result according to the cutoff value of the system; however the identification to the complex level was documented in order to compare the performances of the two systems accurately.

### 2.4. Bruker Microflex LT System

A portion or the whole colony was directly transferred onto the 96 spotted polished steel target plate. The spot was covered with 1 *μ*L of CHCA matrix solution. After completely air drying, the plate was loaded to the Microflex LT instrument. The spectra were generated in linear positive ion mode with delayed extraction in a mass range of 4 to 10 kDa using a 337 nm nitrogen laser with a frequency of 60 Hz. The automated data analysis was processed with MALDI Biotyper Realtime Classification and Biotyper software version 3.0 (Bruker Daltonics). The obtained spectra were analyzed by standard pattern-matching algorithm, which compared the raw spectra with the spectra of the Bruker library by using the standard setting, and the results were listed in a ranking table. The results were expressed as log (score) values, which ranged from 0 to 3 as recommended by the manufacturer. Score values of >1.7 generally indicated relationships at the genus level, and values of >2.0 generally indicated relationships at the species level. The highest score was used for species identification. The Bruker library contains > 80,000 spectra covering 2,048 species and 385 genera. If the result was below 1.7 then the extraction method was performed where the colony was transferred into 1.5-mL screw cap tubes and mixed thoroughly in 0.3 mL of double-distilled ultrapure water. 0.9 mL of pure ethanol was added to tubes and after vortexing they were centrifuged at 13,000 ×g for 2 min. The supernatant was discarded and the pellet was mixed thoroughly with 50 *μ*L of 70% aqueous formic acid. After the addition of 50 *μ*L of acetonitrile, the mixture was centrifuged at 13,000 ×g for 2 min. One microliter of the microorganism extract supernatant was placed onto the polished steel and covered with 1 *μ*L of CHCA matrix solution and loaded to the instrument.

### 2.5. Discordant Results

Results obtained from VITEK MS and Microflex LT were compared to initial onsite phenotypic identification results. The decision matrix used for the comparison and classification of results is given in Table 1. Briefly, when MS and the onsite phenotypic identification results agreed at the species level, then this was accepted as a correct result at the species level. When any MS equipment gave a correct result but as a species complex, then this was considered as a correct result and specified in the result table. For any discordant with the onsite phenotypic identification result we performed a second phenotypic identification. If this second phenotypic identification agreed with the MS result then this second result is accepted depending on correctness level, species, or genus. Otherwise a 16S or ITS1/2 sequencing analysis was performed depending on the nature of microorganism (prokaryote or eukaryote).

### 2.6. Extraction and Purification of Genomic DNA

Three to four colonies were selected from agar plates, resuspended in 500 *μ*L 1x PBS (pH 7.4), and used for genomic DNA isolation. Genomic DNA isolation from Gram-negative bacteria was performed using DNA4U Bacterial Genomic DNA Isolation Kit (NANObiz, Turkey) as instructed by the manufacturer. DNA isolation from Gram-positive bacteria and other microorganisms were performed using conventional phenol/chloroform extraction method. Briefly, samples in 500 *μ*L 1x PBS were centrifuged at 12,500 g for 5 minutes, resuspended in sterile water, recentrifuged, and resuspended in 500 *μ*L TES buffer (50 mM Tris-HCl, pH 8.0; 1 mM EDTA; 6.7% sucrose). For Gram-positive bacteria 75 *μ*L of 10 mg/mL lysozyme was added and samples were incubated at 37°C for 1 hour. After the incubation 125 *μ*L of 20% SDS was added into each sample and incubated at 37°C for 30 minutes. Later 700 *μ*L of phenol (SIGMA, USA) was added and samples were gently mixed using a vortex. Samples were then centrifuged at 2,000 rpm for 5 minutes and aqueous phases were removed into clean microcentrifuge tubes. Equal amount of 24 : 1 (v/v) chloroform : isoamyl alcohol was added and centrifuged at 2,000 rpm for 5 minutes. Aqueous phase was again removed into a clean microcentrifuge tube, and first 0.1 volume of ice cold 3 M potassium acetate, pH 5.5, and then 2 volumes of ice cold absolute ethanol were added. Samples were then incubated at −80°C for 15 minutes. Ethanol was removed through centrifugation at 10,000 rpm for 15 minutes, and this ethanol wash was repeated once more. Obtained pellets were then incubated at 50°C for approximately 15 minutes. Dry pellets were resuspended in 50 *μ*L of sterile water. Purified genomic DNA was either visualized via agarose gel electrophoresis or directly measured for absorbance at 260 nm.

### 2.7. PCR Amplification of 16S Regions

The forward primer S-D-Bact-0008-a-S-20 (5′-AGA GTT TGA TCC TGG CTC AG-3′) [16] and the reverse primer rP2 (5′-ACG GCT ACC TTG TTA CGA CTT-3′) [17], which target the domain Bacteria, were used to amplify bacterial 16S rDNA sequences by PCR. Reaction mixture for PCR contained 1x PCR buffer (Fermentas), 2.0 mM MgCl2, 200 *μ*M dNTP (each), 200 ng template genomic DNA, 0.5 *μ*M of each primer, and 1.0 U Taq polymerase (Fermentas). DNA denaturation and amplification cycles were performed as described [16] using GeneAmp PCR System 9700 thermocycler (Applied Biosystems). Amplification products were checked via agarose gel electrophoresis.

### 2.8. PCR Amplification of ITS Regions

ITS1 (5′-TCCGTAGGTGAACCTGCGG-3′) and ITS4 (5′-TCCTCCGCTTATTGATATGC-3′) primers were used to amplify internal transcribed spacer regions 1 and 2 of microorganisms [18]. Reaction mixture for PCR contained 1x PCR buffer (Fermentas), 1.5 mM MgCl_2_, 100 *μ*M dNTP (each), 0.5 *μ*M of each primer, and 1.0 U Taq polymerase (Fermentas) to a final concentration of 15 *μ*L. DNA denaturation and amplification cycles were performed as described [18].

### 2.9. DNA Sequencing and Sequence Analyses

PCR products were sequenced via Sanger sequencing using 3130 Genetic Analyzer (Applied Biosystems). Obtained sequences were compared to 16S rDNA and/or ITS sequences that are found in public databases: NCBI (http://blast.ncbi.nlm.nih.gov/) and Green Genes 16S rDNA (http://greengenes.lbl.gov/).

## 3. Results

### 3.1. Reference Microorganisms

Eighteen reference bacterial strains were tested (Table 2) for verification and comparison of both MALDI-TOF MS systems.* Salmonella typhimurium* (ATCC 202165),* Salmonella enteritidis* (DM10), and* Salmonella enterica* subsp.* enterica* serovar typhimurium (ATCC 14028) were identified only to the genus level as* Salmonella* spp. These were all expected as they are within the current MALDI-TOF system limitations [1]. The rest of the reference microorganisms could all be accurately identified to the species level (Table 2).

### 3.2. Routine Clinical Bacterial Isolates

Our results indicated that both MS systems had high accuracy in the identification of routine clinical bacterial isolates that cover a wide range of species known to cause human infections (Tables 3 and 4). VITEK MS correctly identified 916 (77.56%) clinical bacterial isolates to the species and 231 (19.56%) isolates to the clinically relevant species group, totaling 1,147 (97.12%) correct identification on the species level. From the same pool, Bruker Microflex LT identified 1,142 (96.70%) clinical isolates to the species level.

Identification on genus only level was also similar for both systems. VITEK MS could identify 19 (1.61%) and Bruker Microflex LT could identify 28 (2.37%) isolates only to the genus level. Genus level, but not to species level identification of* Salmonella* spp. (*n* = 15) accounts for the main decrease in the percentage for correct species level identification for both systems, which is a known system limitation.

Misidentification numbers were similar for VITEK MS and Bruker Microflex LT with 5 (0.42%) and 4 (0.33%) isolates, respectively.* Shigella boydii* was incorrectly identified as* E. coli* by both systems, but this is also a known system limitation.

Except one* Leuconostoc mesenteroides* isolate, the remaining misidentified isolates were identified by at least one system.* L. mesenteroides* was misidentified as* Staphylococcus pettenkoferi* by Bruker Microflex LT, whereas it could not be identified by VITEK MS.

A total of three isolates,* Wautersiella falsenii*,* Kluyvera cryocrescens*, and* Corynebacterium glaucum*, could not be identified by both systems (Table 4).

### 3.3. Fungal Samples

We have observed a very similar identification accuracy in both systems. Out of 160 fungal samples VITEK MS correctly identified 155 (96.88%) isolates to the species, 1 (0.62%) isolate to the species complex, and 3 (1.88%) isolates to the genus level. Similarly, Bruker Microflex LT identified 156 (97.50%) fungal isolates to the species level and 4 (2.50%) isolates to the genus level.

There was no misidentification in both systems. VITEK MS could not identify an* Aspergillus brasiliensis* isolate as this microorganism is not present in the system's library, whereas Bruker Microflex LT identified this isolate to the species level.

## 4. Discussion

Our results indicated that, using any of the two available MALDI-TOF MS systems, a species level correct identification accuracy of over 96% could be achieved without any prior information about the tested samples. We have reached this high accuracy level using the regular sample extraction methods without any strain specific pretreatment in order to determine the efficiency of MS systems in the routine clinical microbiology settings.

In our laboratory, conventional biochemistry based phenotypic microbial identification methods costs about $15 and typically takes from 6 to 18 hours depending on the tested microorganisms. MALDI-TOF MS system, on the other hand, requires only 5 to 30 minutes for the identification and it costs less than $1 per sample.

For this study we collected various clinical specimens which were sent to our laboratory with an initial on-site identification that had been carried out using a VITEK II Compact phenotypic identification platform. During our analyses; for 97 (7.23%) isolates both MS systems gave a different result than this initial on-site phenotypic identification. Our further molecular (16S or ITS1/2 sequencing) or phenotypic analysis resulted in favor of MS systems for all of these 97 isolates. Additional 17 (0.01%) isolates were also confirmed to be different than the on-site phenotypic identification and these isolates were misidentified at least by one of the MS systems. As a result, a total of 114 (8.50%) isolates were misidentified by phenotypic methods, whereas an average of 37 (2.76%) isolates were misidentified or could not be identified by MS systems used in this study, making them at least twice as more accurate (Table 6). Using the wrong ID card and in some few instances wrong labeling might account for these incorrect on-site identifications. MS systems, as they do not need prior strain information, proved themselves more accurate and efficient for this instance.

Identification of microorganisms using a MALDI-TOF system is based on prerecorded protein spectra that are present in the system library [19]. These spectra are mainly based on ribosomal proteins and therefore MALDI-TOF systems have intrinsic limitations to differentiate closely related species or strains such as* Salmonella* spp. In our study the number of identification on the genus only level was 19 (1.61%) for VITEK MS and 28 (2.37%) for Bruker Microflex LT, mainly due to this systems' limitations. Genus level, but not to species level, identification of* Salmonella* spp. (*n* = 15) accounts for the main decrease in the percentage for species level correct identification for both systems.

When we compared the identification accuracies of the two systems we have seen similar results. However we have observed a relatively higher number of correct genera but wrong species count for the Bruker Microflex LT especially with closely related genera or complex members. VITEK MS prefer to give multiple results as species complexes leading to fewer errors while dealing with close species like* Streptococcus parasanguinis/mitis/oralis*,* Enterobacter cloacae/asburiae*,* Citrobacter freundii/youngae*,* Aeromonas hydrophila/caviae*, and* Burkholderia cepacia/vietnamiensis*. However, Bruker Microflex LT tends to report single species and that was the reason of correct genus but wrong species results in 8 cases. This grouping style favors VITEK MS during the analysis of clinical samples because distinguishing these closely related genera is clinically irrelevant; however, it can cause certain limitations for research studies and for the analysis of environmental, food, and industrial samples.

Two* Corynebacterium* spp. (*C. ureicelerivorans* and* C. appendicis*) isolates confirmed with 16S rDNA sequencing could only be correctly identified to the genus level by both systems. Both systems identified this isolate as* C. pseudodiphtericum*. In general, these are rare clinical isolates where molecular identification is needed mainly due to the MALDI-TOF MS library limitations [20]. One* C. ureicelerivorans* and one* Actinomyces naeslundii* isolates, which could not be identified by VITEK MS, were identified to the genus only level by Bruker Microflex LT. Two* Staphylococcus* species* S. simulans* and* S. pettenkoferi* were identified to the genus level by VITEK MS; however, Bruker Microflex LT could identify both of them to the species level. On the other hand, one* Myroides odoratimimus* isolate could only be identified to genus level by Bruker Microflex LT, whereas it was identified to species level by VITEK MS.

VITEK MS had a relatively higher no-identification rate compared to Bruker Microflex LT in the Coryneforms and other Gram-positive bacilli group (Table 5). It has already been shown that probably due to the thick peptidoglycan layer, as it might interfere with the laser ionization, better results had been obtained with a pretreatment prior to MS run for Gram-positive bacteria [21].

Among the 3 species that could not be identified by both systems (Table 4)* W. falsenii* was not included in the VITEK MS library both in genus and in species level.* C. glaucum* was represented on the genus level but not on the species level in the VITEK MS library.* K. cryocrescens*, on the other hand, was already in the library. As we had only one isolate for the MALDI-TOF analysis, it could have been a strain specific issue. Bruker had spectra for all these three species in its library.* W. falsenii* and* C. glaucum* are represented with 2 spectra, whereas* K. cryocrescens* is represented with only 1 spectrum in Bruker BioTyper library. It is likely that this low number of spectral representation in the library was the reason for no identification. In the upcoming updates once their spectral representation is increased or included in the library, we believe both systems will perform better in the identification.

Evaluating all results together, we have observed that most of the species with incorrect and/or no identifications were not common microorganisms encountered in clinical settings and also they are either not represented in systems' libraries or represented with low number of spectra. We believe that these issues will be solved once systems' libraries are updated in the near future.

VITEK MS' species complex approach (reporting the closely related species together), which is a guaranteed attitude, increases the rate of correct identification and it is an advantage for the identifications from the routine clinical isolates. Bruker Microflex LT, however, confidently acts and reports single species, which can provide an advantage in research studies as well as in analyses of environmental, food, and industrial samples. However, consequently, both systems correctly identified 97% of routine clinical isolates making them reliable tools for clinical microbiology laboratories.

Comparing final confirmed identifications, MALDI-TOF MS systems showed a better identification accuracy compared to phenotypic identification. In addition to a better accuracy, a faster workflow and considerable lower cost provide clinical laboratories with a better identification tool [22, 23].

## Figures and Tables

**Table 1:** Decision table used for the classification of results during the study.

Type	On-site phenotypic identification	MALDI-TOF MS analysis		Secondary phenotypic identification or molecular identification (16S or ITS1/2 sequencing)	Accepted	Number of m/o's (*n* = 1341^*∗*^)
		VITEK	Bruker			
A	Identified as *X*	Identified as *X*	Identified as *X*	N/A	Accepted as *X*	1203

B	Identified as *X* or no-identification	Identified as *Y*	Identified as *Y*	Identified as *Y*	Accepted as *Y*	97

C	Identified as *X*	Identified as *X*	Identified as *Y*	Identified as *X* (or *Y*)	Accepted as *X* (or *Y*)	16
		Identified as *Y*	Identified as *X*			

D	Identified as *X*	Identified as *Y*	Identified as *Z*	Identified as *Y* (or *Z*) (sequencing only)	Accepted as *Y* (or *Z*)	6

E	Identified as *X*	Identified as *Y*	Identified as *Y*	Identified as *Z* (sequencing only)	Accepted as *Z*	3

F	Identified as *X*	No-identification	Identified as *X* (or *Y*)	Identified as *X* (or *Y* or *Z*)	Accepted as *X* (or *Y* or *Z*)	12
		Identified as *X* (or *Y*)	No-identification			

G	Identified as *X*	No-identification	No-identification	Identified as *X* (or *Y*) (sequencing only)	Accepted as *X* (or *Y*)	3

^*∗*^ *Shigella boydii* was not included.

**Table 2:** Results of 18 reference bacterial strains that were analyzed by VITEK MS and Bruker Microflex LT for an initial system setup and comparison.

Microorganism	VITEK	Bruker
*S. pyogenes* ATCC 19615	✓	✓

*E. coli* ATCC 25922	✓	✓

*K. pneumoniae* ATCC 13883	✓	✓

*P. aeruginosa* ATCC 27853	✓	✓

*S. aureus* ATCC 25923	✓	✓

*S. pneumoniae* ATCC 49619	✓	✓

*P. vulgaris* ATCC 13315	*P. vulgaris/penneri*	*P. hauseri* *(new nomenclature)*

*S. typhimurium* ATCC 14028	*Salmonella group*	*Salmonella *spp.

*Y. enterocolitica* ATCC 9610	✓	✓

*V. choler*a RSKK 913	✓	✓ (*V. cholerae *biovar albensis)

*G. vaginalis* DM 108	✓	✓

*S. saprophyticus* DM 17	✓	✓

*S. agalactiae* DM 20	✓	✓

MRSA DM 66	*S. aureus*	*S. aureus*

*S. epidermidis* DM 19	✓	✓

*S. enteritidis* DM 10	*Salmonella *group	*Salmonella* spp.

*A. hydrophila* DM 110	*A. hydrophila/caviae*	*A. caviae*

*P. shigelloides* DM 111	✓	✓

**Table 3:** Summary of high-confidence identifications by VITEK MS and Bruker Microflex LT MS systems.

Microorganism groups	Number of isolates	Species content	Correct Identification				Misidentification		No-identification	
			Species		Genus only					
			VITEK	Bruker	VITEK	Bruker	VITEK	Bruker	VITEK	Bruker
Nonfermentatives	356 (30.1%)	16	352 (98.8%)	350 (98.4%)	—	3 (0.8%)	2 (0.6%)	—	2 (0.6%)	3 (0.8%)
Enterobacteriaceae	309 (26.2%)	23	292 (94.5%)	288 (93.2%)	15 (4.9%)	19 (6.2%)	1 (0.3%)	1 (0.3%)	1 (0.3%)	1 (0.3%)
Staphylococci	273 (23.1%)	10	271 (99.3%)	270 (98.9%)	2 (0.7%)	—	—	2 (0.7%)	—	1 (0.4%)
Streptococci	29 (2.5%)	11	28 (96.6%)	27 (93.2%)	—	1 (3.4%)	—	1 (3.4%)	1 (3.4%)	—
Enterococci and other Gr(+) cocci	160 (13.6%)	5	160 (100.0%)	160 (100.0%)	—	—	—	—	—	—
Coryneforms and other Gr(+) bacilli	30 (2.5%)	14	21 (70.00%)	25 (83.3%)	2 (6.7%)	4 (13.4%)	2 (6.7%)	—	5 (16.6%)	1 (3.3%)
HACEK and other Gr(−) cocci	18 (1.5%)	8	18 (100.0%)	18 (100.0%)	—	—	—	—	—	—
Anaerobes	2 (0.2%)	2	2 (100.0%)	2 (100.0%)	—	—	—	—	—	—
Others	4 (0.3%)	4	3 (75.0%)	2 (50.0%)	—	1 (25.0%)	—	—	1 (25.0%)	1 (25.0%)
TOTAL (bacteria)	**1,181**	**93**	**1,147 (97.1%)**	**1,142 (96.7%)**	**19 (1.6%)**	**28 (2.4%)**	**5 (0.4%)**	**4 (0.3%)**	**10 (0.9%)**	**7 (0.6%)**
Fungi	160	14	156 (97.5%)	156 (97.5%)	3 (1.9%)	4 (2.5%)	—	—	1 (0.6%)	—
TOTAL (bacteria + fungi)	**1,341**	**107**	**1,303 (97.2%)**	**1,298 (96.8%)**	**22 (1.6%)**	**32 (2.4%)**	**5 (0.4%)**	**4 (0.3%)**	**11 (0.8%)**	**7 (0.5%)**

**Table 4:** High-confidence identifications by VITEK MS and Bruker Microflex LT MS systems.

Microorganism groups	# of isolates	Correct Identification					Misidentification		No-identification	
		Species			Genus only					
		VITEK		Bruker	VITEK	Bruker	VITEK	Bruker	VITEK	Bruker
		*Species*	*Species Complex*							
Nonfermentatives *(16 species)*										
*Achromobacter xylosoxidans*	6	4	2	6	—	—	—	—	—	—
*Acinetobacter baumannii*	194	—	192	192	—	—	—	—	2 (F × 2)	2 (F × 2)
*Acinetobacter guillouiae*	1	—	1	1	—	—	—	—	—	—
*Acinetobacter johnsonii*	1	1	—	1	—	—	—	—	—	—
*Acinetobacter lwoffii*	1	1	—	1	—	—	—	—	—	—
*Acinetobacter radioresistens*	1	1	—	1	—	—	—	—	—	—
*Aeromonas hydrophila*	3	—	3	2	—	1 (C)	—	—	—	—
*Aeromonas sobria*	1	—	1	—	—	—	—	—	—	1 (F)
*Alcaligenes faecalis*	2	2	—	2	—	—	—	—	—	—
*Burkholderia cepacia*	2	—	2	—	—	2 (C × 2)	—	—	—	—
*Comamonas aquatica*	1	—	—	1	—	—	1 (D)	—	—	—
*Delftia acidovorans*	3	3	—	3	—	—	—	—	—	—
*Elizabethkingia meningoseptica*	1	1	—	1	—	—	—	—	—	—
*Pseudomonas aeruginosa*	125	124	—	125	—	—	1 (C)	—	—	—
*Pseudomonas fluorescens*	1	1	—	1	—	—	—	—	—	—
*Stenotrophomonas maltophilia*	13	13	—	13	—	—	—	—	—	—
Total	**356**	**151 ** **(42.4%)**	**201 (56.4%)**	**350 (98.4%)**	**—**	**3 (0.8%)**	**2 (0.6%)**	**—**	**2** ** (0.6%)**	**3** ** (0.8%)**

Enterobacteriaceae *(23 species)*										
*Chryseobacterium indologenes*	1	1	—	1	—	—	—	—	—	—
*Citrobacter braakii*	3	—	3	3	—	—	—	—	—	—
*Citrobacter freundii*	5	3	2	5	—	—	—	—	—	—
*Citrobacter sedlakii*	1	1	—	1	—	—	—	—	—	—
*Citrobacter youngae*	2	—	2	1	—	1 (C)	—	—	—	—
*Enterobacter aerogenes*	4	4	—	4	—	—	—	—	—	—
*Enterobacter asburiae*	2	—	2	1	—	1 (C)	—	—	—	—
*Enterobacter cancerogenous*	1	1	—	1	—	—	—	—	—	—
*Enterobacter cloacae*	11	—	11	9	—	2 (C × 2)	—	—	—	—
*Escherichia coli*	123	123	—	123	—	—	—	—	—	—
*Klebsiella oxytoca*	5	5	—	5	—	—	—	—	—	—
*Klebsiella pneumonia*	64	64	—	64	—	—	—	—	—	—
*Kluyvera cryocrescens*	1	—	—	—	—	—	—	—	1 (G)	1 (G)
*Morganella morganii*	12	12	—	12	—	—	—	—	—	—
*Proteus mirabilis*	28	28	—	28	—	—	—	—	—	—
*Providencia rettgeri*	3	3	—	3	—	—	—	—	—	—
*Providencia stuartii*	1	1	—	1	—	—	—	—	—	—
*Raoultella ornithinolytica*	3	2	1	3	—	—	—	—	—	—
*Raoultella planticola*	1	—	1	1	—	—	—	—	—	—
*Salmonella *spp.	15	—	—	—	15	15	—	—	—	—
*Serratia liquefaciens*	1	1	—	1	—	—	—	—	—	—
*Serratia marcescens*	21	21	—	21	—	—	—	—	—	—
*Shigella boydii*	1	—	—	—	—	—	1 (N/A)	1 (N/A)	—	—
Total	**309**	**270 ** **(87.4%)**	**22 (7.1%)**	**288 (93.3%)**	**15 (4.9%)**	**19 (6.1%)**	**1 (0.3%)**	**1** **(0.3%)**	**1** ** (0.3%)**	**1** **(0.3%)**

Staphylococci *(10 species)*										
*Staphylococcus aureus*	52	52	—	52	—	—	—	—	—	—
*Staphylococcus capitis*	8	8	—	8	—	—	—	—	—	—
*Staphylococcus caprae*	1	1	—	1	—	—	—	—	—	—
*Staphylococcus epidermidis*	68	68	—	65	—	—	—	2 (C × 2)	—	1 (F)
*Staphylococcus haemolyticus*	54	54	—	54	—	—	—	—	—	—
*Staphylococcus hominis*	82	82	—	82	—	—	—	—	—	—
*Staphylococcus lugdunensis*	1	1	—	1	—	—	—	—	—	—
*Staphylococcus pettenkoferi*	1	—	—	1	1 (C)	—	—	—	—	—
*Staphylococcus sciuri*	2	2	—	2	—	—	—	—	—	—
*Staphylococcus simulans*	4	3	—	4	1 (D)	—	—	—	—	—
Total	**273**	**271 ** **(99.3%)**	**—**	**270 (98.9%)**	**2 (0.7%)**	**—**	**—**	**2** **(0.7%)**	**—**	**1** ** (0.4%)**

Streptococci *(11 species)*										
*Leuconostoc mesenteroides*	1	—	—	—	—	—	—	1 (F)	1 (F)	—
*Streptococcus agalactiae*	9	9	—	9	—	—	—	—	—	—
*Streptococcus anginosus*	1	1	—	1	—	—	—	—	—	—
*Streptococcus dysgalactiae*	3	3	—	3	—	—	—	—	—	—
*Streptococcus gallolyticus*	1	1	—	1	—	—	—	—	—	—
*Streptococcus mitis*	1	—	1	—	—	1 (C)	—	—	—	—
*Streptococcus oralis*	1	—	1	1	—	—	—	—	—	—
*Streptococcus parasanguinis*	1	1	—	1	—	—	—	—	—	—
*Streptococcus pneumonia*	4	4	—	4	—	—	—	—	—	—
*Streptococcus pyogenes*	6	6	—	6	—	—	—	—	—	—
*Streptococcus salivarius*	1	1	—	1	—	—	—	—	—	—
Total	**29**	**26 ** **(89.7%)**	**2 (6.9%)**	**27 (93.2%)**	**—**	**1 (3.4%)**	**—**	**1** ** (3.4%)**	**1** ** (3.4%)**	**—**

Enterococci and other Gr(+) cocci *(5 species)*										
*Aerococcus viridans*	4	4	—	4	—	—	—	—	—	—
*Enterococcus avium*	4	4	—	4	—	—	—	—	—	—
*Enterococcus faecalis*	54	54	—	54	—	—	—	—	—	—
*Enterococcus faecium*	84	84	—	84	—	—	—	—	—	—
*Enterococcus gallinarum*	14	14	—	14	—	—	—	—	—	—
Total	**160**	**160 ** **(100%)**	**—**	**160 (100%)**	**—**	**—**	**—**	**—**	**—**	**—**

Coryneforms and other Gr(+) bacilli *(14 species)*										
*Actinomyces naeslundii*	1	—	—	—	—	1 (F)	—	—	1 (F)	—
*Bacillus cereus*	1	—	1	1	—	—	—	—	—	—
*Bacillus simplex*	1	1	—	1	—	—	—	—	—	—
*Corynebacterium amycolatum*	4	—	4	4	—	—	—	—	—	—
*Corynebacterium appendicis*	1	—	—	—	1 (E)	1 (E)	—	—	—	—
*Corynebacterium coyleae*	1	—	—	1	—	—	1 (D)	—	—	—
*Corynebacterium glaucum*	1	—	—	—	—	—	—	—	1 (G)	1 (G)
*Corynebacterium jeikeium*	2	2	—	2	—	—	—	—	—	—
*Corynebacterium mucifaciens*	3	—	—	3	—	—	1 (D)	—	2 (F)	—
*Corynebacterium striatum*	10	10	—	10	—	—	—	—	—	—
*Corynebacterium urealyticum*	1	1	—	1	—	—	—	—	—	—
*Corynebacterium ureicelerivorans*	2	—	—	—	1 (E)	2 (E, F)	—	—	1 (F)	—
*Lactobacillus paracasei*	1	—	1	1	—	—	—	—	—	—
*Listeria monocytogenes*	1	1	—	1	—	—	—	—	—	—
Total	**30**	**15 ** **(50.0%)**	**6 (20.0%)**	**25 (83.4%)**	**2 (6.7%)**	**4 (13.3%)**	**2 (6.7%)**	**—**	**5** ** (16.6%)**	**1** ** (3.3%)**

HACEK and other Gr(−) cocci *(8 species)*										
*Haemophilus haemolyticus*	1	1	—	1	—	—	—	—	—	—
*Haemophilus influenza*	3	3	—	3	—	—	—	—	—	—
*Haemophilus parahaemolyticus*	2	2	—	2	—	—	—	—	—	—
*Haemophilus parainfluenzae*	1	1	—	1	—	—	—	—	—	—
*Legionella pneumophila*	7	7	—	7	—	—	—	—	—	—
*Moraxella catarrhalis*	1	1	—	1	—	—	—	—	—	—
*Neisseria meningitides*	1	1	—	1	—	—	—	—	—	—
*Pasteurella multocida*	2	2	—	2	—	—	—	—	—	—
Total	**18**	**18 ** **(100%)**	**—**	**18 (100%)**	**—**	**—**	**—**	**—**	**—**	**—**

Anaerobes *(2 species)*										
*Clostridium perfringens*	1	1	—	1	—	—	—	—	—	—
*Clostridium sporogenes*	1	1	—	1	—	—	—	—	—	—
Total	**2**	**2 ** **(100%)**	**—**	**2 (100%)**	**—**	**—**	**—**	**—**	**—**	**—**

Others *(4 species)*										
*Bergeyella zoohelcum*	1	1	—	1	—	—	—	—	—	—
*Myroides odoratimimus*	1	1	—	—	—	1 (C)	—	—	—	—
*Rhodococcus equi*	1	1	—	1	—	—	—	—	—	—
*Wautersiella falsenii*	1	—	—	—	—	—	—	—	1 (G)	1 (G)
Total	**4**	**3 ** **(75.0%)**	**—**	**2 (50.0%)**	**—**	**1 (C) (25.0%)**	**—**	**—**	**1 (G)** ** (25.0%)**	**1 (G) ** **(25.0%)**

Total (bacteria)	**1,181**	**916 (77.6%)**	**231 (19.6%)**	**1,142 (96.7%)**	**19 (1.6%)**	**28 (2.4%)**	**5 (0.4%)**	**4** ** (0.3%)**	**10** **(0.8%)**	**7** **(0.6%)**

Fungi *(14 species)*										
*Aspergillus brasiliensis*	1	—	—	1	—	—	—	—	1 (F)	—
*Candida albicans*	43	43	—	41	—	2 (C × 2)	—	—	—	—
*Candida dubliniensis*	1	1	—	1	—	—	—	—	—	—
*Candida glabrata*	44	44	—	44	—	—	—	—	—	—
*Candida kefyr*	11	11	—	11	—	—	—	—	—	—
*Candida krusei*	4	4	—	4	—	—	—	—	—	—
*Candida lusitaniae*	2	2	—	2	—	—	—	—	—	—
*Candida norvegensis*	1	1	—	1	—	—	—	—	—	—
*Candida orthopsilosis*	2	—	—	2	2 (D × 2)	—	—	—	—	—
*Candida parapsilosis*	14	14	—	13	—	1 (C)	—	—	—	—
*Candida tropicalis*	32	31	1	32						
*Cryptococcus neoformans*	1	1	—	1	—	—	—	—	—	—
*Meyerozyma caribbica*	1	—	—	—	1 (E)	1 (E)	—	—	—	—
*Trichosporon asahii*	3	3	—	3	—	—	—	—	—	—
Total (fungi)	**160**	**155 ** **(96.9%)**	**1 (0.6%)**	**156 (97.5%)**	**3 (1.9%)**	**4 (2.5%)**	**—**	**—**	**1** **(0.6%)**	**—**

TOTAL (bacteria + fungi)	**1,341**	**1,071 (79.9%)**	**232 (17.3%)**	**1,298 (96.8%)**	**22 (1.6%)**	**32 (2.4%)**	**5 (0.4%)**	**4** **(0.3%)**	**11 (0.8%)**	**7** **(0.5%)**
		**1,303 (97.2%)**								

**Table 5:** Results summary of MALDI-TOF MS systems for the microorganisms that either were misidentified or could not be identified.

Microorganism groups	Number of isolates	Species content	Misidentification		No-identification	
			VITEK	Bruker	VITEK	Bruker
Nonfermentatives						
*Acinetobacter baumannii*	194		—	—	2 (F × 2)	2 (F × 2)
*Aeromonas sobria*	1		—	—	—	1 (F)
Total	**356**	**16**	**2**	**—**	**2**	**3**

Enterobacteriaceae						
*Kluyvera cryocrescens*	1		—	—	1 (G)	1 (G)
*Shigella boydii*	1		1 (N/A)	1 (N/A)	—	—
Total	**309**	**23**	**1**	**1**	**1**	**1**

Staphylococci						
*Staphylococcus epidermidis*	68		—	2 (C × 2)	—	1 (F)
Total	**273**	**10**	**—**	**2**	**—**	**1**

Streptococci						
*Leuconostoc mesenteroides*	1		—	1 (F)	1 (F)	—
Total	**29**	**11**	**—**	**1**	**1**	**—**

Enterococci and other Gr(+) cocci						
Total	**160**	**5**	**—**	**—**	**—**	**—**

Coryneforms and other Gr(+) bacilli						
*Actinomyces naeslundii*	1		—	—	1 (F)	—
*Corynebacterium coyleae*	1		1 (D)	—	—	—
*Corynebacterium glaucum*	1		—	—	1 (G)	1 (G)
*Corynebacterium mucifaciens*	3		1 (D)	—	2 (F)	—
*Corynebacterium ureicelerivorans*	2		—	—	1 (F)	—
Total	**30**	**14**	**2**	**—**	**5**	**1**

HACEK and other Gr(−) cocci						
Total	**18**	**8**	**—**	**—**	**—**	**—**

Anaerobes						
Total	**2**	**2**	**—**	**—**	**—**	**—**

Others						
*Wautersiella falsenii*	1		—	—	1 (G)	1 (G)
Total	**4**	**4**	**—**	**—**	**1 (G)**	**1 (G)**

Total (bacteria)	**1,181**	**93**	**5**	**4**	**10**	**7**

Fungi						
*Aspergillus brasiliensis*	1		—	—	1 (F)	—
Total (fungi)	**160**	**14**	**—**	**—**	**1**	**—**

Total (bacteria + fungi)	**1,341**	**107**	**5**	**4**	**11**	**7**

**Table 6:** Detailed results of microorganisms that were either misidentified or could not be identified by at least one MALDI-TOF system or phenotypic identification.

Correct ID	Number of isolates	On-site phenotypic identification	Secondary phenotypic identification or molecular identification^*∗*^ (16S or ITS1/2 sequencing)	VITEK	Bruker
Nonfermentatives					
*Achromobacter xylosoxidans*	1	***Achromobacter denitrificans***	^*∗*^ *Achromobacter xylosoxidans*	*Achromobacter denitrificans/xylosoxidans*	*Achromobacter xylosoxidans*
*Acinetobacter baumannii*	1	***Cedecea lapegei***	*Acinetobacter baumannii*	*Acinetobacter baumannii *complex	*Acinetobacter baumannii*
*Acinetobacter baumannii*	1	***Escherichia coli***	*Acinetobacter baumannii *complex	*Acinetobacter baumannii *complex	*Acinetobacter baumannii*
*Acinetobacter baumannii*	1	***Klebsiella pneumoniae***	*Acinetobacter baumannii*	*Acinetobacter baumannii *complex	*Acinetobacter baumannii*
*Acinetobacter baumannii*	1	***Pseudomonas aeruginosa***	*Acinetobacter baumannii*	*Acinetobacter baumannii *complex	*Acinetobacter baumannii*
*Acinetobacter baumannii*	1	***Staphylococcus hominis***	*Acinetobacter baumannii *complex	*Acinetobacter baumannii *complex	*Acinetobacter baumannii*
*Acinetobacter baumannii*	2	*Acinetobacter baumannii*	*Acinetobacter baumannii*	**No identification**	*Acinetobacter baumannii*
*Acinetobacter baumannii*	2	*Acinetobacter baumannii*	*Acinetobacter baumannii*	*Acinetobacter baumannii complex*	**No identification**
*Acinetobacter guillouiae*	1	***Acinetobacter lwoffii***	^*∗*^ *Acinetobacter guillouiae*	***Acinetobacter baumannii complex***	*Acinetobacter guillouiae*
*Acinetobacter johnsonii*	1	***Acinetobacter lwoffii***	^*∗*^ *Acinetobacter johnsonii*	*Acinetobacter johnsonii*	*Acinetobacter johnsonii*
*Acinetobacter radioresistens*	1	***Acinetobacter lwoffii***	^*∗*^ *Acinetobacter radioresistens*	*Acinetobacter radioresistens*	*Acinetobacter radioresistens*
*Aeromonas hydrophila*	1	***Aeromonas sobria***	^*∗*^ *Aeromonas hydrophila*	*Aeromonas hydrophila/caviae*	*Aeromonas hydrophila*
*Aeromonas hydrophila*	1	*Aeromonas hydrophila/caviae*	*Aeromonas hydrophila*	*Aeromonas hydrophila/caviae*	***Aeromonas caviae***
*Aeromonas sobria*	1	*Aeromonas sobria*	^*∗*^ *Aeromonas sobria*	*Aeromonas hydrophila/caviae, sobria*	**No identification**
*Alcaligenes faecalis*	1	***Chromobacterium violaceum***	^*∗*^ *Alcaligenes faecalis*	*Alcaligenes faecalis*	*Alcaligenes faecalis*
*Burkholderia cepacia*	2	*Burkholderia cepacia*	^*∗*^ *Burkholderia cepacia*	*Burkholderia cepacia/vietnamiensis*	***Burkholderia cenocepacia***
*Comamonas aquatica*	1	***Comamonas testosteroni***	^*∗*^ *Comamonas aquatic*	***Delftia acidovorans***	*Comamonas aquatica*
*Delftia acidovorans*	1	***No identification***	*Delftia acidovorans*	*D. acidovorans*	*D. acidovorans*
*Delftia acidovorans*	1	***No identification***	*Delftia acidovorans*	*Delftia acidovorans*	*Delftia acidovorans*
*Pseudomonas aeruginosa*	3	***Acinetobacter baumannii***	*Pseudomonas aeruginosa*	*Pseudomonas aeruginosa*	*Pseudomonas aeruginosa*
*Pseudomonas aeruginosa*	1	***Enterobacter aerogenes***	*Pseudomonas aeruginosa*	*Pseudomonas aeruginosa*	*Pseudomonas aeruginosa*
*Pseudomonas aeruginosa*	1	*Pseudomonas aeruginosa*	*Pseudomonas aeruginosa*	***Porphyromonas gingivalis***	*Pseudomonas aeruginosa*
*Stenotrophomonas maltophilia*	1	***Pseudomonas aeruginosa***	*Stenotrophomonas maltophilia*	*Stenotrophomonas maltophilia*	*Stenotrophomonas maltophilia*

Enterobacteriaceae					
*Chryseobacterium indologenes*	1	**No identification**	*Chryseobacterium indologenes*	*Chryseobacterium indologenes*	*Chryseobacterium indologenes*
*Citrobacter braakii*	1	***Citrobacter freundii***	^*∗*^ *Citrobacter braakii*	*Citrobacter braakii/farmeri/freundii*	*Citrobacter braakii*
*Citrobacter freundii*	1	**No identification**	*Citrobacter freundii*	*Citrobacter freundii*	*Citrobacter freundii*
*Citrobacter sedlakii*	1	***Citrobacter amalonaticus***	^*∗*^ *Citrobacter sedlakii*	*Citrobacter sedlakii*	*Citrobacter sedlakii*
*Citrobacter youngae*	1	***No identification***	*Citrobacter youngae*	*Citrobacter freundii/youngae*	*Citrobacter youngae*
*Citrobacter youngae*	1	*Citrobacter youngae*	^*∗*^ *Citrobacter youngae*	*Citrobacter freundii/youngae*	***Citrobacter freundii***
*Enterobacter asburiae*	1	***Escherichia coli***	*Enterobacter cloacae *complex	*Enterobacter cloacae/asburiae*	*Enterobacter asburiae*
*Enterobacter asburiae*	1	***Enterobacter cloacae *complex**	^*∗*^ *Enterobacter asburiae*	*Enterobacter cloacae/asburiae*	***Enterobacter cloacae***
*Enterobacter cancerogenous*	1	***Enterobacter aerogenes***	*Enterobacter cancerogenous*	*Enterobacter cancerogenous*	*Enterobacter cancerogenous*
*Enterobacter cloacae*	1	***Enterobacter cancerogenus***	*Enterobacter cloacae *complex	*Enterobacter cloacae/asburiae*	*Enterobacter cloacae*
*Enterobacter cloacae*	1	**No identification**	*Enterobacter cloacae *complex	*Enterobacter cloacae/asburiae*	*Enterobacter cloacae*
*Enterobacter cloacae*	1	**No identification**	*Enterobacter cloacae*	*Enterobacter cloacae/asburiae*	*Enterobacter cloacae*
*Enterobacter cloacae*	1	*Enterobacter cloacae*	^*∗*^ *Enterobacter cloacae*	*Enterobacter cloacae/asburiae*	***Enterobacter asburiae***
*Enterobacter cloacae*	1	*Enterobacter cloacae*	*Enterobacter cloacae*	*Enterobacter cloacae/asburiae*	***Enterobacter asburiae***
*Enterobacter cloacae*	1	**No identification**	*Enterobacter cloacae*	*Enterobacter cloacae/asburiae*	*Enterobacter cloacae*
*Klebsiella oxytoca*	1	***Escherichia coli***	*Klebsiella oxytoca*	*Klebsiella oxytoca*	*Klebsiella oxytoca*
*Klebsiella pneumoniae*	1	***Acinetobacter baumannii***	*Klebsiella pneumonia*	*Klebsiella pneumoniae*	*Klebsiella pneumoniae*
*Klebsiella pneumoniae*	1	***Enterobacter aerogenes***	*Klebsiella pneumonia*	*Klebsiella pneumoniae*	*Klebsiella pneumoniae*
*Klebsiella pneumoniae*	1	***Enterobacter aerogenes***	^*∗*^ *Klebsiella pneumoniae*	*Klebsiella pneumoniae*	*Klebsiella pneumoniae*
*Klebsiella pneumoniae*	1	***Escherichia coli***	*Klebsiella pneumonia*	*Klebsiella pneumoniae*	*Klebsiella pneumoniae*
*Kluyvera cryocrescens*	1	***Raoultella planticola***	^*∗*^ *Kluyvera cryocrescens*	**No identification**	**No identification**
*Proteus mirabilis*	1	***Stenotrophomonas maltophilia***	*Proteus mirabilis*	*Proteus mirabilis*	*Proteus mirabilis*
*Raoultella ornithinolytica*	1	**No identification**	^*∗*^ *Raoultella ornithinolytica*	*Raoultella ornithinolytica/planticola*	*Raoultella ornithinolytica*
*Shigella boydii*	1	*Shigella boydii*	*Shigella boydii*	***Escherichia coli***	***Escherichia coli***

Staphylococci					
*Staphylococcus aureus*	1	***Acinetobacter baumannii***	*Staphylococcus aureus*	*Staphylococcus aureus*	*Staphylococcus aureus*
*Staphylococcus hominis*	1	*Acinetobacter baumannii*	*Staphylococcus hominis*	*Staphylococcus hominis*	*Staphylococcus hominis*
*Staphylococcus epidermidis*	1	***Enterococcus faecium***	*Staphylococcus epidermidis*	*Staphylococcus epidermidis*	*Staphylococcus epidermidis*
*Staphylococcus simulans*	1	***Granulicatella elegans***	^*∗*^ *Staphylococcus simulans*	*Staphylococcus haemolyticus*	*Staphylococcus simulans*
*Staphylococcus haemolyticus*	1	***Staphylococcus aureus***	*Staphylococcus haemolyticus*	*Staphylococcus haemolyticus*	*Staphylococcus haemolyticus*
*Staphylococcus pettenkoferi*	1	***Staphylococcus auricularis***	^*∗*^ *Staphylococcus pettenkoferi*	***Staphylococcus auricularis/capitis***	*Staphylococcus pettenkoferi*
*Staphylococcus hominis*	1	***Staphylococcus epidermidis***	*Staphylococcus hominis*	*Staphylococcus hominis*	*Staphylococcus hominis*
*Staphylococcus aureus*	1	***Staphylococcus haemolyticus***	*Staphylococcus aureus*	*Staphylococcus aureus*	*Staphylococcus aureus*
*Staphylococcus capitis*	1	***Staphylococcus haemolyticus***	*Staphylococcus capitis*	*Staphylococcus capitis*	*Staphylococcus capitis*
*Staphylococcus aureus*	1	***Staphylococcus hominis***	*Staphylococcus aureus*	*Staphylococcus aureus*	*Staphylococcus aureus*
*Staphylococcus epidermidis*	1	***Staphylococcus hominis***	*Staphylococcus epidermidis*	*Staphylococcus epidermidis*	*Staphylococcus epidermidis*
*Staphylococcus epidermidis*	1	***Staphylococcus lentus***	*Staphylococcus epidermidis*	*Staphylococcus epidermidis*	*Staphylococcus epidermidis*
*Staphylococcus hominis*	1	***Staphylococcus saprophyticus***	*Staphylococcus hominis*	*Staphylococcus hominis*	*Staphylococcus hominis*
*Staphylococcus hominis*	1	***Staphylococcus warneri***	^*∗*^ *Staphylococcus hominis*	*Staphylococcus hominis*	*Staphylococcus hominis*
*Staphylococcus hominis*	2	***Staphylococcus warneri***	^*∗*^ *Staphylococcus hominis*	*Staphylococcus hominis*	*Staphylococcus hominis*
*Staphylococcus haemolyticus*	1	***Staphylococcus warneri***	*Staphylococcus haemolyticus*	*Staphylococcus haemolyticus*	*Staphylococcus haemolyticus*
*Staphylococcus epidermidis*	1	*Staphylococcus epidermidis*	*Staphylococcus epidermidis*	*Staphylococcus epidermidis*	***Bacillus pumilus***
*Staphylococcus epidermidis*	1	*Staphylococcus epidermidis*	*Staphylococcus epidermidis*	*Staphylococcus epidermidis*	***Dermobacter hominis***
*Staphylococcus aureus*	1	**No identification**	*Staphylococcus aureus*	*Staphylococcus aureus*	*Staphylococcus aureus*
*Staphylococcus lugdunensis*	1	**No identification**	*Staphylococcus lugdunensis*	*Staphylococcus lugdunensis*	*Staphylococcus lugdunensis*
*Staphylococcus epidermidis*	1	*Staphylococcus epidermidis*	*Staphylococcus epidermidis*	*Staphylococcus epidermidis*	**No identification**

Streptococci					
*Leuconostoc mesenteroides*	1	***Staphylococcus capitis***	^*∗*^ *Leuconostoc mesenteroides*	**No identification**	***Staphylococcus pettenkoferi***
*Streptococcus agalactiae*	3	**No identification**	*Streptococcus agalactiae*	*Streptococcus agalactiae*	*Streptococcus agalactiae*
*Streptococcus dysgalactiae*	1	**No identification**	^*∗*^ *Streptococcus dysgalactiae*	*Streptococcus dysgalactiae*	*Streptococcus dysgalactiae*
*Streptococcus mitis*	1	*Streptococcus mitis*	*Streptococcus mitis*	*Streptococcus parasanguinis/mitis/oralis*	***Streptococcus pneumoniae***
*Streptococcus salivarius*	1	***Streptococcus lentus***	*Streptococcus salivarius*	*Streptococcus salivarius*	*Streptococcus salivarius*

Enterococci & other Gr(+) cocci					
*Enterococcus faecalis*	1	***Escherichia coli***	*Enterococcus faecalis*	*Enterococcus faecalis*	*Enterococcus faecalis*
*Enterococcus faecium*	1	***Enterococcus faecalis***	*Enterococcus faecium*	*Enterococcus faecium*	*Enterococcus faecium*
*Enterococcus faecalis*	2	***Enterococcus faecium***	*Enterococcus faecalis*	*Enterococcus faecalis*	*Enterococcus faecalis*
*Enterococcus gallinarum*	1	***Enterococcus faecium***	^*∗*^ *Enterococcus gallinarum*	*Enterococcus gallinarum*	*Enterococcus gallinarum*
*Enterococcus faecium*	2	***Enterococcus gallinarum***	*Enterococcus faecium*	*Enterococcus faecium*	*Enterococcus faecium*
*Enterococcus faecalis*	1	***Serratia fonticola***	*Enterococcus faecalis*	*Enterococcus faecalis*	*Enterococcus faecalis*
*Enterococcus faecalis*	1	***Staphylococcus hominis***	*Enterococcus faecalis*	*Enterococcus faecalis*	*Enterococcus faecalis*

Coryneforms & other Gr(+) bacilli					
*Corynebacterium striatum*	1	***Actinomyces spp.***	*Corynebacterium striatum*	*Corynebacterium striatum*	*Corynebacterium striatum*
*Corynebacterium urealyticum*	1	***Brucella spp.***	*Corynebacterium urealyticum*	*Corynebacterium urealyticum*	*Corynebacterium urealyticum*
*Corynebacterium appendicis*	1	***Corynebacterium jeikeium***	^*∗*^ *Corynebacterium appendicis*	***Corynebacterium pseudodiphtericum***	***Corynebacterium pseudodiphtericum***
*Corynebacterium ureicelerivorans*	1	***Corynebacterium jeikeium***	^*∗*^ *Corynebacterium ureicelerivorans*	***Corynebacterium afermentans***	***Corynebacterium afermentans***
*Corynebacterium mucifaciens*	2	***Corynebacterium jeikeium***	^*∗*^ *Corynebacterium mucifaciens*	**No identification**	*Corynebacterium mucifaciens*
*Corynebacterium ureicelerivorans*	1	***Corynebacterium jeikeium***	^*∗*^ *Corynebacterium ureicelerivorans*	**No identification**	***Corynebacterium coyleae***
*Corynebacterium glaucum*	1	***Corynebacterium jeikeium***	^*∗*^ *Corynebacterium glaucum*	**No identification**	**No identification**
*Corynebacterium mucifaciens*	1	***Clostridium *spp.**	^*∗*^ *Corynebacterium mucifaciens*	***Rhizobium radiobacter***	*Corynebacterium mucifaciens*
*Corynebacterium coyleae*	1	***Dermococcus/Kytococcus/*** ***Micrococcus***	^*∗*^ *Corynebacterium coyleae*	***Arthrobacter cumminsii***	*Corynebacterium coyleae*
*Lactobacillus paracasei*	1	***Pediococcus pentosaceus***	^*∗*^ *Lactobacillus paracasei*	*Lactobacillus casei/paracasei*	*Lactobacillus paracasei*
*Corynebacterium striatum*	1	***Propionibacterium acnes***	*Corynebacterium striatum*	*Corynebacterium striatum*	*Corynebacterium striatum*
*Bacillus simplex*	1	***Sphingomonas paucimobilis***	*Bacillus simplex*	*Bacillus simplex*	*Bacillus simplex*
*Corynebacterium amycolatum*	1	***Bacillus *spp.**	*Corynebacterium amycolatum*	*Corynebacterium amycolatum/xerosis*	*Corynebacterium amycolatum*
*Corynebacterium jeikeium*	2	***Bacillus *spp.**	*Corynebacterium jeikeium*	*Corynebacterium jeikeium*	*Corynebacterium jeikeium*
*Corynebacterium striatum*	2	***Bacillus *spp.**	*Corynebacterium striatum*	*Corynebacterium striatum*	*Corynebacterium striatum*
*Actinomyces naeslundii*	1	*Actinomyces naeslundii*	^*∗*^ *Actinomyces naeslundii*	**No identification**	*Actinomyces *spp.

HACEK & other Gr(−) cocci					
*Moraxella catarrhalis*	1	***Arcanobacterium hemoliticum***	*Moraxella catarrhalis*	*Moraxella catarrhalis*	*Moraxella catarrhalis*
*Haemophilus influenzae*	1	***Bacillus *spp.**	*Haemophilus influenzae*	*Haemophilus influenzae*	*Haemophilus influenzae*
*Pasteurella multocida*	1	***Bacillus *spp.**	^*∗*^ *Pasteurella multocida*	*Pasteurella multocida*	*Pasteurella multocida*

Others					
*Bergeyella zoohelcum*	1	**No identification**	^*∗*^ *Bergeyella zoohelcum*	*Bergeyella zoohelcum*	*Bergeyella zoohelcum*
*Wautersiella falsenii*	1	***Weeksella virosa***	^*∗*^ *Wautersiella falsenii*	**No identification**	**No identification**
*Myroides odoratimimus*	1	*Myroides *spp.	^*∗*^ *Myroides odoratimimus*	*Myroides odoratimimus*	*Myroides *spp.

Fungi					
*Candida glabrata*	2	***Candida albicans***	*Candida glabrata*	*Candida glabrata*	*Candida glabrata*
*Candida albicans*	1	***Candida famata***	*Candida albicans*	*Candida albicans*	*Candida albicans*
*Candida tropicalis*	1	***Candida famata***	*Candida tropicalis*	*Candida tropicalis/glabrata*	*Candida tropicalis*
*Candida tropicalis*	1	***Candida famata***	^*∗*^ *Candida tropicalis*	*Candida tropicalis*	*Candida tropicalis*
*Candida albicans*	2	***Candida glabrata***	*Candida albicans*	*Candida albicans*	*Candida albicans*
*Candida glabrata*	1	***Candida lusitaniae***	*Candida glabrata*	*Candida glabrata*	*Candida glabrata*
*Candida tropicalis*	2	***Candida parapsilosis***	*Candida tropicalis*	*Candida tropicalis*	*Candida tropicalis*
*Candida glabrata*	1	***Candida rugose***	*Candida glabrata*	*Candida glabrata*	*Candida glabrata*
*Candida kefyr*	1	***Candida sphaerica***	*Candida kefyr*	*Candida kefyr*	*Candida kefyr*
*Candida orthopsilosis*	1	***Candida tropicalis***	^*∗*^ *Candida orthopsilosis*	***Candida parapsilosis***	*Candida orthopsilosis*
*Candida parapsilosis*	2	*Candida *spp.	^*∗*^ *Candida parapsilosis*	*Candida parapsilosis*	*Candida parapsilosis*
*Candida orthopsilosis*	1	*Candida *spp.	^*∗*^ *Candida orthopsilosis*	***Candida parapsilosis***	*Candida orthopsilosis*
*Meyerozyma caribbica*	1	*Candida *spp.	^*∗*^ *Meyerozyma caribbica*	***Candida guilliermondii***	***Candida guilliermondii***
*Candida albicans*	1	***Stephanoascus ciferrii***	*Candida albicans*	*Candida albicans*	*Candida albicans*
*Candida krusei*	1	**No identification**	*Candida krusei*	*Candida krusei*	*Candida krusei*
*Candida albicans*	1	*Candida albicans*	*Candida albicans*	*Candida albicans*	***Candida tropicalis***
*Candida albicans*	1	*Candida albicans*	*Candida albicans*	*Candida albicans*	***Candida glabrata***
*Candida parapsilosis*	1	*Candida parapsilosis*	^*∗*^ *Candida parapsilosis*	*Candida parapsilosis*	***Candida orthopsilosis***
*Aspergillus brasiliensis*	1	*Aspergillus brasiliensis*	*Aspergillus brasiliensis*	**No identification**	*Aspergillus brasiliensis*

^*∗*^Molecular identification (16S or ITS1/2 sequencing).
